# Accessible interview practices for disabled scientists and engineers

**DOI:** 10.1016/j.isci.2024.110220

**Published:** 2024-06-26

**Authors:** Samuel M. Greene, Sandra R. Schachat, Naomi Arita-Merino, Xiangkun Elvis Cao, Harsha Gurnani, Michael Heyns, Maria L. Cagigas, Caitlin L. Maikawa, Elise J. Needham, Ethan A. Perets, Elizabeth Phillips, Anthony W. Waddle, Christine E. Wilkinson, Kevin C. Zhou, Hannah M. Zlotnick

**Affiliations:** 1 Oden Institute for Computational Engineering and Sciences, The University of Texas at Austin, Austin, TX 78712, USA; 2 Entomology Section, Department of Plant and Environmental Sciences, The University of Hawai’i at Mānoa, Honolulu, Hawai’i 96822, USA; 3 Sustainable Food Processing Laboratory, Institute of Food, Nutrition and Health, ETH Zürich, 8092 Zürich, Switzerland; 4 Department of Chemical Engineering, Massachusetts Institute of Technology, Cambridge, MA 02139, USA; 5 Department of Biology, University of Washington, Seattle, WA, USA; 6 Blackett Laboratory, Imperial College London, London, UK; 7 Sydney Medical School Nepean, University of Sydney, Sydney, NSW, Australia; 8 Institute of Biomedical Engineering, University of Toronto, Toronto, ON, Canada; 9 British Heart Foundation Cardiovascular Epidemiology Unit, Department of Public Health and Primary Care, University of Cambridge, Cambridge, UK; 10 Victor Phillip Dahdaleh Heart and Lung Research Institute, University of Cambridge, Cambridge, UK; 11 Department of Molecular Biology, University of Texas Southwestern Medical Center, Dallas, TX, USA; 12 Inorganic Chemistry Laboratory, University of Oxford, Oxford, UK; 13 Applied Biosciences, Macquarie University, Sydney, NSW 2109, Australia; 14 Department of Environmental Science, Policy, and Management, University of California, Berkeley, Berkeley, CA, USA; 15 California Academy of Sciences, San Francisco, CA, USA; 16 Department of Electrical Engineering and Computer Sciences, University of California, Berkeley, Berkeley, CA, USA; 17 BioFrontiers Institute, University of Colorado, Boulder, CO 80303, USA

## Abstract

Increasing representation of people with disabilities in science and engineering will require systemic changes to the culture around support and accommodations. Equitable interview practices can help foster such changes. We, an interdisciplinary group of disabled and nondisabled early-career scientists who care deeply about making science more accessible to all, present a framework of suggestions based on Universal Design principles for improving the accessibility and equitability of interviews for people with disabilities and other underrepresented groups. We discuss potential challenges that may arise when implementing these suggestions and provide questions to guide discussions about addressing them.

## Graphical abstract

**Figure:**
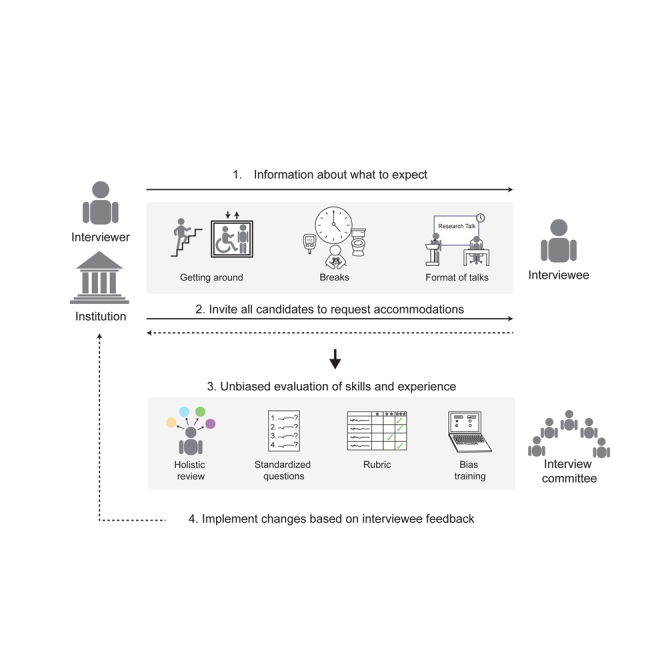

**Figure:**
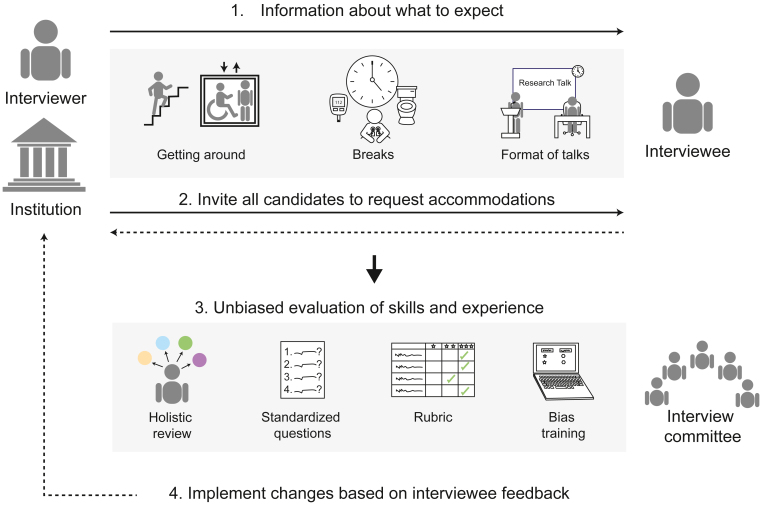
Above image: Our proposed strategies for implementing more accessible and equitable interview processes

Reducing the challenges faced by people with disabilities in science and engineering will require large-scale systemic changes—perhaps most foundationally, to people’s perceptions of disabilities and the culture around supporting people with disabilities.

Making interviews more accessible is a particularly important step toward improving disability culture, as an interview is often an applicant’s first encounter with a new work environment*.*

Ensuring that people with disabilities can fully participate in science and engineering, including in leadership positions, will accelerate scientific discovery and benefit society*.*

### Introduction

People with disabilities are underrepresented throughout science and engineering. In the United States, disabled scientists become more underrepresented at each successive stage of education. An estimated 21% of people aged 18–44 have at least one disability.^1^ But among recipients of degrees in science and engineering, only 12%, 10%, and 8% of those receiving bachelor’s, master’s, and doctoral degrees, respectively, reported a disability.^2^ Doctorate recipients with disabilities are underrepresented among faculty with tenure, deans, and university presidents, and their annual salaries are less than those of nondisabled doctoral degree recipients.^3^ Underrepresentation is by no means limited to the United States. In the United Kingdom, for example, people with disabilities are 50% less likely to have jobs in science and engineering than their nondisabled counterparts.^4^ Data related to people with disabilities are collected and reported less frequently than for other demographic groups, making it difficult to diagnose and therefore address this underrepresentation.

Systemic challenges at multiple levels contribute to underrepresentation of people with disabilities. At an interpersonal level, some people may fear being viewed as less capable or credible because of their disability^5^ and as a result decide not to pursue advanced studies or a career in the sciences. Anecdotes from scientists with disabilities indicate that such concerns sometimes reflect real biases that can adversely affect science research and education (Box 1). Encountering people with disabilities may make some people uncomfortable and therefore unwilling to engage them in substantive scientific discussions or consider them for positions.^6^ At an institutional level, features of laboratories and other professional scientific environments can make certain tasks difficult for researchers with disabilities. For example, many laboratories cannot be easily navigated with a wheelchair, crutches, or a cane, and many tasks involve standing for long periods of time.^7^ Current accommodation mechanisms often do not adequately address these issues.^8^ Even when accommodations are approved, they are sometimes difficult to access. Scientists with non-obvious or undiagnosed disabilities may hesitate to request accommodations and therefore may not receive the support they need to succeed in the sciences (Box 1). At a societal level, the intersection of disability with other marginalized identities compounds these challenges. For example, people of color with disabilities report more financial barriers to accessing healthcare than non-Hispanic white people with disabilities,^9^ which can complicate the process of substantiating requests for accommodations.

Box 1 **Personal anecdotes from some of the authors illustrate the challenges that people with disabilities face while pursuing careers in science and engineering**

(1) I often question my decision not to disclose my disability when joining a new lab. I think that once others know me and my work, it will be safe to share this information and request the accommodations I need, because I have proved I am worth it. I feel guilty for hiding my disability and frustrated because I could perform better and strain myself less if I had access to accommodations. If I was given the opportunity to openly discuss my disability during an interview or when starting a new job, I would, as it would signal an environment where equity and inclusion are important.
(2) As an undergraduate, I received extended time on written exams due to my disability, as approved by my university’s disability office. One professor told me that I “didn’t deserve” extended time on his written exams, but that he would grant it to me anyway. When I achieved high exam scores, he told me that they were solely a result of my accommodations rather than my studiousness. I did not feel that I could raise this issue with the disability office, as I needed this particular professor to write me a recommendation letter for graduate school and therefore did not want to upset him.

Reducing the challenges faced by people with disabilities in science and engineering will require large-scale systemic changes—perhaps most foundationally, to people’s perceptions of disabilities and the culture around supporting people with disabilities. This article addresses one practice that shapes this culture: interviewing in science and engineering (e.g., for undergraduate internships, PhD programs, fellowships, postdoctoral positions, faculty positions, and employment in industry or government labs). Making interviews more accessible is a particularly important step toward improving disability culture, as an interview is often an applicant’s first encounter with a new work environment. Applicants assess the supportiveness of an environment through their interview experience and may hesitate to raise issues because the stakes are high. In addition to increasing representation of people with disabilities, improving interview practices can foster a culture of support for the successful applicants who join the labs and programs managed by their interviewers.

Interviewees are usually allowed to request accommodations for disabilities (as required by law in many places), but existing accommodations policies often do not adequately address the challenges that people with disabilities face. In some settings, interviewees are not explicitly invited to request accommodations. When such invitations are made, they often reflect only a sentiment of legal obligation rather than a genuine willingness to support interviewees. Poorly phrased invitations may pressure interviewees to disclose sensitive information about their disability unrelated to the accommodations they need, and they may worry that this will adversely affect their perceived competence. In some cases, interviewers presume an understanding of what accommodations are needed. For example, one resource suggests phrasing interview questions such that they can be answered succinctly by interviewees with speech impediments, but such interviewees may instead want to be afforded the patience to discuss their past accomplishments in rich detail. Those who encounter incorrect presumptions may feel reluctant to address them. A different approach to accommodating interviewees is therefore needed.

This article articulates a framework of four suggestions for making the interview process more accessible and equitable. This framework can be applied to a variety of interview settings, including those outside the sciences, and can be adapted as needed. This article is not intended as a criticism of our previous interviewers, many of whom have implemented these practices effectively. Rather, we intend to provoke discussion and thoughtful reflection on the broader culture around disability in science and engineering. The term “disability” encompasses a broad range of experiences, including both obvious and non-obvious disabilities, some of which may be labeled as disabilities in some settings but not in others. Some people may be undecided about whether a challenge they face constitutes a disability. Our recommendations do not rely upon a specific definition of disability and instead reflect the principles of Universal Design, which is the practice of improving accessibility for all, regardless of disability status (Box 2). We therefore expect that our recommendations will also benefit scientists from other underrepresented groups who may face similar challenges in interview settings, including neurodivergent applicants, those from different cultural backgrounds or religions, those with non-binary gender identities, non-native English speakers, and those from disadvantaged socioeconomic backgrounds.

Box 2 **Universal design principles**

Universal Design refers to “the design of products and environments to be usable by all people, to the greatest extent possible, without the need for adaptation or specialized design.”^10^ It was first developed in the context of architecture, but its principles have since been applied in other settings, including education.^11^ There are seven principles guiding users’ interactions with an environment or product; those most relevant to interview settings include^10^:

(1) Equitable use: allowing all users to interact with an environment through similar or equivalent means, without imposing stigmas
(2) Flexibility in use: providing multiple means by which users can interact with an environment
(3) Perceptible information: conveying feedback and information to the user via modes compatible with diverse communication and sensory abilities
(4) Tolerance for error: mitigating adverse consequences of unintended actions

### Suggestion 1: Provide information about what to expect before the interview

The setting of an interview can greatly affect the extent to which people with disabilities experience difficulties. We suggest that interviewers provide information in an accessible format to all interviewees, by default, about the interview setting. It should be provided with sufficient notice to allow accommodations to be arranged (likely at least 1–2 weeks, depending on the setting). This information could relate to the format (e.g., answering questions or giving a presentation, and any associated time constraints), the location(s) (specifically wheelchair accessibility and travel between multiple locations), the seating arrangement (e.g., whether the interviewee will be seated or standing at a podium, and whether a microphone will be used), who will be present, whether food will be served, and the timing and accessibility of breaks (during which interviewees may need, for example, to use the restroom, test their blood sugar, or pump breast milk). Institutions can aggregate and anonymize information about accommodations received by previous successful applicants and then provide it to interviewees to demonstrate that requests for accommodations have not prevented hiring of applicants with disabilities in the past. As appropriate, some interview questions could be provided to all applicants beforehand. In addition to benefiting people with disabilities, these practices can also reduce inequities associated with the fact that some interviewees from privileged backgrounds (e.g., those from elite institutions) may have access to more information about what to expect. All interviewees would benefit from receiving the same information. This suggestion reflects Universal Design principles 1 and 3 (equitable use and perceptible information).

### Suggestion 2: Invite all interviewees to request accommodations, regardless of disability status

After providing information about what to expect, interviewers should initiate a conversation with every interviewee to co-create an interview environment that will meet their needs. Interviewers should avoid pathologizing disability and instead use language consistent with the goal to create a supportive environment, for example, “We want to help you present the best version of yourself in this interview so that we can accurately assess your fit for this position.” Interviewers should demonstrate a willingness to modify the interview schedule and explicitly invite all interviewees to ask questions and request modifications. They could provide examples of the kinds of accommodations that are available (for example, additional breaks, sign language interpretation, improved ventilation, etc.) while emphasizing that this list is not exclusive. Initiating this conversation with *all* interviewees reduces the pressure that those with disabilities may feel to disclose or explain their disability. Additionally, this approach could facilitate efforts to improve the interview process for all. For example, if many interviewees request more time for breaks, interview schedules could be modified to include longer breaks by default. Once a schedule and accommodations are agreed upon, all people involved in the interview should be informed about these accommodations and explicitly asked to adhere to them throughout the interview process. This ensures that interviewees will not encounter unexpected challenges during their interview. This suggestion reflects Universal Design principles 1 and 2 (equitable use and flexibility in use).

### Suggestion 3: Evaluate interviewees’ skills and experiences as they apply to the role in an unbiased manner

Many current approaches to assessing interviewees put those with disabilities at a disadvantage. In some settings, interviewers may subconsciously make incorrect assumptions about a disabled applicant’s capabilities. For example, people with speech impediments are sometimes perceived as less intelligent and less competent,^12^ as are non-native English speakers.^13^ Interview processes that rely too heavily on standardized metrics do not adequately account for the variety of skills and experiences that may have prepared applicants for the position, or for the unique challenges that people with disabilities may have faced. Holistic review, which involves considering the full range of each candidate’s unique attributes when assessing their fit, has been previously shown to address issues like these and increase the diversity of successful applicants.^14^ Holistic review strategies should therefore be used to assess all interviewees. Such strategies include using a rubric, standardizing interview questions, requiring bias training for interviewers, and standardizing methods for ranking interviewees.^14^ The rubric and ranking methods should reflect the specific responsibilities of the position, accommodate multiple means of fulfilling them, and include clear criteria that interviewers can use to assess candidates. Interview questions should reflect the rubric. Asking questions focused on responsibilities will allow interviewees with disabilities to discuss any accommodations they may need to fulfill these responsibilities, thereby preempting incorrect assumptions. Reminding interviewers of the empirical evidence linking such interventions to reductions in implicit bias can help increase buy-in.^15^ Acknowledging the diverse ways in which people can be successful scientists can help address ableist (as well as racist, sexist, and other) attitudes. This suggestion reflects Universal Design principles 1 and 4 (equitable use and tolerance for error).

### Suggestion 4: Seek feedback and implement changes as needed

Establishing an interview framework that suits people with a broad range of abilities and backgrounds is inherently challenging. We therefore recommend that changes be made through an iterative process of feedback-seeking, revision, and refinement. Interviewees may hesitate to provide feedback out of concern that it will influence the outcome of their interview. We therefore recommend that a third party, such as a university-wide accommodations office, collect and anonymize feedback for interviewers. Feedback should be solicited as soon as possible after an interview, when the interviewee can easily recall their experience. The third party could then hold their feedback in escrow until a final decision is made, in order to ensure impartiality. If an interviewee reports a negative experience, the third party could recommend changes to *all* interviewers, in order to avoid revealing that interviewee’s identity. Anonymized feedback should be made available to future applicants to allow them to assess the supportiveness of the working environment and the effectiveness of accommodation policies. Additionally, current employees with disabilities could be asked what policies could have improved their interview experiences, and their unedited responses could be made available to future applicants. These practices will help ensure accountability and transparency.

### Conclusions

This article describes a framework of suggestions for making interviews more equitable and accessible. Implementing this framework is a crucial step toward addressing the systemic underrepresentation of these individuals in science and engineering and will require coordinated efforts from multiple entities. Our recommendations can benefit people from other underrepresented backgrounds as well, as they are derived from Universal Design principles. We provide questions to help guide internal discussions around their implementation (Box 3). However, we also expect that national funding agencies can help address these challenges and reduce redundant efforts across organizations, for example, by providing standardized interview guidelines and requesting evidence of their implementation. We expect that our recommendations alone will be insufficient to engender the change needed to support people with disabilities in science and engineering, so we urge that they be implemented with complementary policies that support this goal. Ensuring that people with disabilities can fully participate in science and engineering, including in leadership positions, will accelerate scientific discovery and benefit society.

Box 3 **Considerations for implementation**

Implementing these suggestions within the context of existing practices and policies will require careful consideration. Here, we provide questions to facilitate efficient discussions and guide effective implementation. These questions may be especially helpful for groups that have not previously selected people with disabilities for a particular role.

(1) How will a list of accommodations received by previous successful applicants be compiled and anonymized?
(2) How will suggestions from current workers with disabilities be solicited and aggregated?
(3) How will interviewees be assured that accommodation requests will not adversely affect their chances?
(4) What questions should be provided before the interview?
(5) What mechanisms or rubrics are needed to ensure a fair, objective selection process?
(6) How will anonymous feedback be collected?
